# Channel-mediated astrocytic glutamate modulates hippocampal synaptic plasticity by activating postsynaptic NMDA receptors

**DOI:** 10.1186/s13041-015-0097-y

**Published:** 2015-02-03

**Authors:** Hyungju Park, Kyung-Seok Han, Jinsoo Seo, Jaekwang Lee, Shashank M Dravid, Junsung Woo, Heejung Chun, Sukhee Cho, Jin Young Bae, Heeyoung An, Woohyun Koh, Bo-Eun Yoon, Rolando Berlinguer-Palmini, Guido Mannaioni, Stephen F Traynelis, Yong Chul Bae, Se-Young Choi, C Justin Lee

**Affiliations:** Center for Neural Science, Korea Institute of Science and Technology (KIST), Seoul, Korea; Neuroscience Program, University of Science and Technology (UST), Daejeon, Korea; Department of Physiology and Dental Research Institute, Seoul National University School of Dentistry, Seoul, Korea; Department of Pharmacology, Creighton University, Omaha, NE USA; Department of Oral Anatomy and Neurobiology, School of Dentistry, Kyungpook National University, Daegu, Korea; KU-KIST Graduate School of Converging Science and Technology, Seoul, Korea; Department of Nanobiomedical Science, Dankook University, Cheonan, Korea; School of Electrical and Electronic Engineering, Institute of Neuroscience, Newcastle University, Newcastle upon Tyne, UK; Department of Pharmacology, University of Florence, Florence, Italy; Department of Pharmacology, Emory University, Atlanta, GA USA

**Keywords:** Astrocytes, Bestrophin 1, Ca^2+^-activated anion channel, Synaptic plasticity, Glutamate, NMDA receptor, LTP, PAR1

## Abstract

**Background:**

Activation of G protein coupled receptor (GPCR) in astrocytes leads to Ca^2+^-dependent glutamate release via Bestrophin 1 (Best1) channel. Whether receptor-mediated glutamate release from astrocytes can regulate synaptic plasticity remains to be fully understood.

**Results:**

We show here that Best1-mediated astrocytic glutamate activates the synaptic N-methyl-D-aspartate receptor (NMDAR) and modulates NMDAR-dependent synaptic plasticity. Our data show that activation of the protease-activated receptor 1 (PAR1) in hippocampal CA1 astrocytes elevates the glutamate concentration at Schaffer collateral-CA1 (SC-CA1) synapses, resulting in activation of GluN2A-containing NMDARs and NMDAR-dependent potentiation of synaptic responses. Furthermore, the threshold for inducing NMDAR-dependent long-term potentiation (LTP) is lowered when astrocytic glutamate release accompanied LTP induction, suggesting that astrocytic glutamate is significant in modulating synaptic plasticity.

**Conclusions:**

Our results provide direct evidence for the physiological importance of channel-mediated astrocytic glutamate in modulating neural circuit functions.

## Background

Growing evidence has supported the idea that astrocytes are actively involved in modulating synaptic strength by affecting neuronal properties [1-3]. At glutamatergic synapses, astrocytically released glutamate has been suggested to play a crucial role in mediating neuronal-glial circuits. Astrocytes not only clear presynaptically released glutamates during synaptic transmission, but can also release glutamate via diverse pathways such as soluble NSF attachment protein receptor (SNARE)-dependent exocytosis [4-6] and the glutamate permeable anion channel [7-11], in response to increased intracellular Ca^2+^ concentration by activation of G-protein coupled receptors expressed at the astrocytic membrane. In turn, this Ca^2+^-dependent glutamate release from astrocytes can be sensed by presynaptic or postsynaptic glutamate receptors such as the metabotropic glutamate receptor (mGluR) [6,12] or NMDAR [13,14], both of which are known to modify presynaptic and postsynaptic activities, or synaptic plasticity.

However, the role of astrocytes in synaptic function is still in question, because recent studies have given contradictory reports of the involvement of receptor-mediated Ca^2+^ signals in astrocytic glutamate release [15-18]. In order to pinpoint the exact role of astrocytic glutamate in synaptic functions, we thus have searched for an effective and reliable tool for triggering glutamate release from astrocytes. Mounting evidence supports the ability of PAR1 to trigger the Ca^2+^-dependent signaling pathways crucial for astrocytic glutamate release. Activated by endogenous agonist (thrombin, plasmin) or TFLLR-NH_2_ peptide agonist (TFLLR) [19,20], PAR1 can elevate astrocytic intracellular Ca^2+^ levels via downstream pathways associated with Ca^2+^ release from internal stores [19,20]. In addition, PAR1 activation was shown to be effective for triggering Ca^2+^-dependent astrocytic glutamate release when compared with the activation by other GPCRs [14,21]. Furthermore, due to selective and functional expression of PAR1 in astrocytes in the hippocampal CA1 area [20,22], PAR1 signaling has the additional advantage of inducing astrocyte-specific Ca^2+^-dependent signaling in the hippocampal CA1 area without affecting pre- or postsynaptic neurons. Therefore, PAR1 appears to be a useful tool for selective induction of Ca^2+^-dependent glutamate release from astrocytes *in vitro* and *in vivo* [10,11,14,20,23-26].

Our previous studies have shown that PAR1 activation in hippocampal CA1 astrocytes leads to Ca^2+^-dependent opening of the glutamate-permeable anion channel, Best1, which mediates Ca^2+^-dependent astrocytic glutamate release [8,10,11,26]. Not only does the Best1 channel displays a glutamate permeability that is Ca^2+^-dependent [8], but it also has a preferential subcellular localization at the microdomains of hippocampal astrocytes located around synaptic terminals [10]. These studies suggest that PAR1-induced Ca^2+^ elevation at the microdomain directs glutamate release through the Best1 channel, resulting in an increase in glutamate concentration at synaptic clefts. Moreover, Best1-mediated astrocytic glutamate release triggered by PAR1 activation may play a role in modulating in synaptic plasticity, as recent studies show that PAR1-deficient mice display reduced NMDAR-dependent hippocampal LTP and contextual fear memory [27].

To explore the target and physiological consequences of Best1-mediated glutamate release from astrocytes, we triggered Ca^2+^-dependent glutamate release from hippocampal CA1 astrocytes by activating PAR1, and examined the effect of astrocytic glutamate on neurotransmission. We demonstrated that synaptic NMDAR is the main target of astrocytic Best1-mediated glutamate, and increased synaptic NMDAR activation leads to NMDAR-dependent potentiation of synaptic transmission. Of equally importance, we also identified an altered NMDAR-dependent synaptic plasticity at hippocampal synapses, when synaptic glutamate was increased by Best1-mediated secretion of glutamate from astrocytes. As well as verifying the functional expression of the mechanism for receptor-mediated glutamate release in astrocytes, our findings provide direct evidence for the involvement of astrocytic anion channel-mediated glutamate release in synaptic modification.

## Results

### Astrocytes release glutamate via Best1 channel upon PAR1 activation

**Figure 1:**
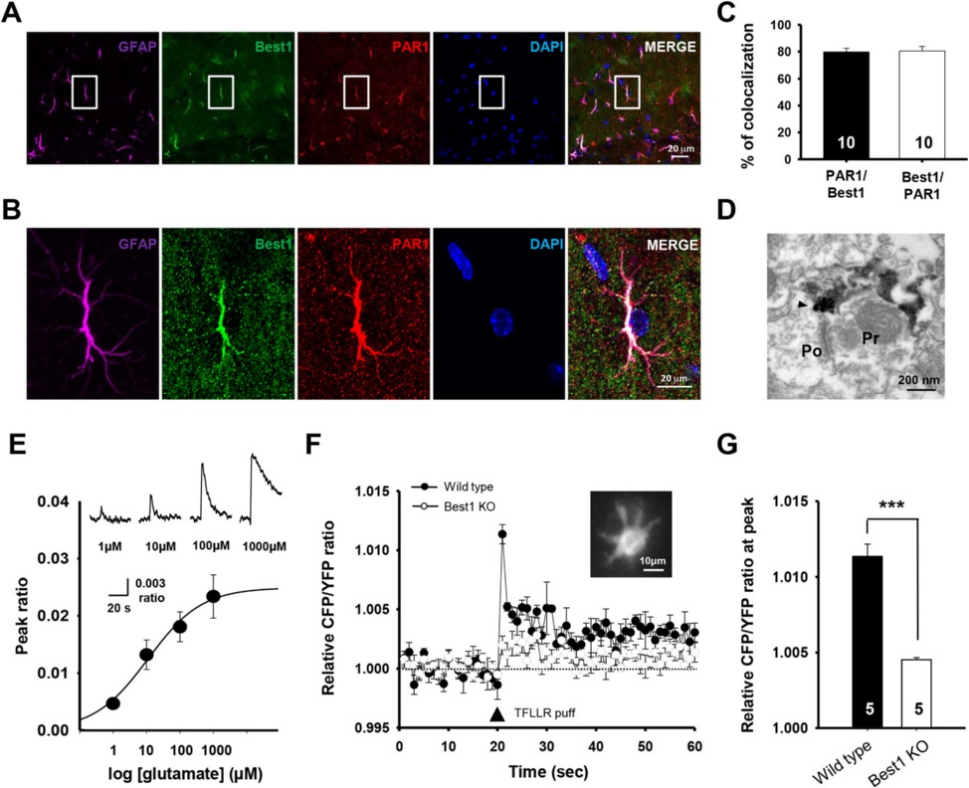
**PAR1 activation induces astrocytic glutamate release via Best1. A**, Immunohistochemistry images showing endogenous expression patterns of GFAP (magenta), Best1 (green), PAR1 (red), and nucleus (DAPI in blue) in hippocampal CA1 area. **B**, Magnified views of the yellow box in Figure 1A. **C**, Bar graphs represent the percentage of PAR1-expressing astrocytes among astrocytes showing Best1 expression (PAR1/Best1) or Best1-expressing astrocytes among astrocytes showing PAR1 expression (Best1/PAR1). Mean ± standard error (s.e.m). Numbers of astrocytes analyzed are indicated within bars. **D**, Representative electron microscopy image showing localization of endogenous Best1 in hippocampal CA1 astrocytes. Po, postsynaptic terminal; Pr, presynaptic terminal. Arrowhead indicates Best1 staining at astrocytic microdomain near synaptic terminals. **E**, ***Above***, representative GluSFnR (shown as relative CFP/YFP ratio) in response to bath application of indicated glutamate concentration. ***Below***, concentration-effect curve representing the averaged peak CFP/YFP ratio (mean ± s.e.m.) induced by application of glutamate at various concentrations. **F**, Graph showing the averaged relative CFP/YFP ratio values (mean ± s.e.m.) from time-lapse imaging of GluSFnR-expressing astrocytes in hippocampal CA1 area of wild type (black) and *Best1* knockout (KO) mice (red). Arrowhead indicates the time at which TFLLR puff (30 μM; 500 ms) was applied. *Inset:* a representative GluSFnR-expressing hippocampal astrocyte in hippocampal slices. **G**, Bar graph represents averaged peak amplitudes of relative CFP/YFP ratio (mean ± s.e.m.) measured from GluSFnR-expressing astrocytes in hippocampal CA1 area of wild type (black) or *Best1* knockout (KO) mice. ***, P < 0.005, unpaired two-tailed Student’s t-test. Numbers of tested slices from at least three independent mice are indicated within each bar.

We firstly observed the expression pattern of endogenous PAR1 at hippocampal Schaffer collateral pathways (SC-CA1 synapses). Immunostaining analysis using PAR1-specific antibody showed that endogenous PAR1 is selectively expressed in astrocytes, because ~90% of GFAP-positive astrocytes showed PAR1 expression (90.0 ± 4.9%, n = 4), whereas there was no significant expression in neuronal cells, as shown in previous studies in human and rat brain (Figure 1A,B) [20,22].

Of more importance, immunohistochemical analysis by co-staining endogenous PAR-1 and Best1 proteins in the CA1 area, showed that both PAR1 and Best1 are highly co-localized in CA1 astrocytes (PAR1/Best1: 79.8 ± 1.6%, n = 10; Best1/PAR1: 80.5 ± 1.6%, n = 10; Figure 1C). Because the astrocytic Best1 channel is localized at the microdomain of astrocytic processes near the synaptic region (Figure 1D) [10], and Ca^2+^-activated Best1 channel showed a significant permeability to glutamate in hippocampal astrocytes [11], our finding raises a possibility that glutamate release through Best1 channel at astrocytic microdomains could affect synaptic glutamate concentration.

To directly test whether PAR1 activation can induce astrocytic glutamate release through Best1 channel, we monitored extracellular glutamate by using fluorescence resonance energy transfer (FRET)-based glutamate sensor GluSnFR (a glutamate-sensing fluorescent reporter) [28]. This GluSnFR was expressed at the membrane of CA1 astrocytes in hippocampal slices to detect glutamate released from astrocytes. Control experiments showed that astrocytic GluSnFR sensors were able to detect extracellular glutamate in a range from 10^−3^ to 10^−6^ M (Figure 1E), similar to that found in cultured astrocytes [26]. We found that bath application of the PAR1 agonist, TFLLR (30 μM) [8,20,26] increases extracellular glutamate level (at the peak; 8.5 ± 1.9 μM, n = 5) around a single CA1 astrocyte, and that this elevation of extracellular glutamate level was significantly reduced in slices of *Best1* knockout mice (Best1 KO : Figure 1F,G). In line with previous findings [8,10,11,26], these results indicate that PAR1 activation-triggered astrocytic glutamate release is mediated by Best1 channels, which possibly permeate intracellular glutamate into extracellular synaptic clefts.

### Best1-mediated astrocytic glutamate enhances basal synaptic transmission

**Figure 2:**
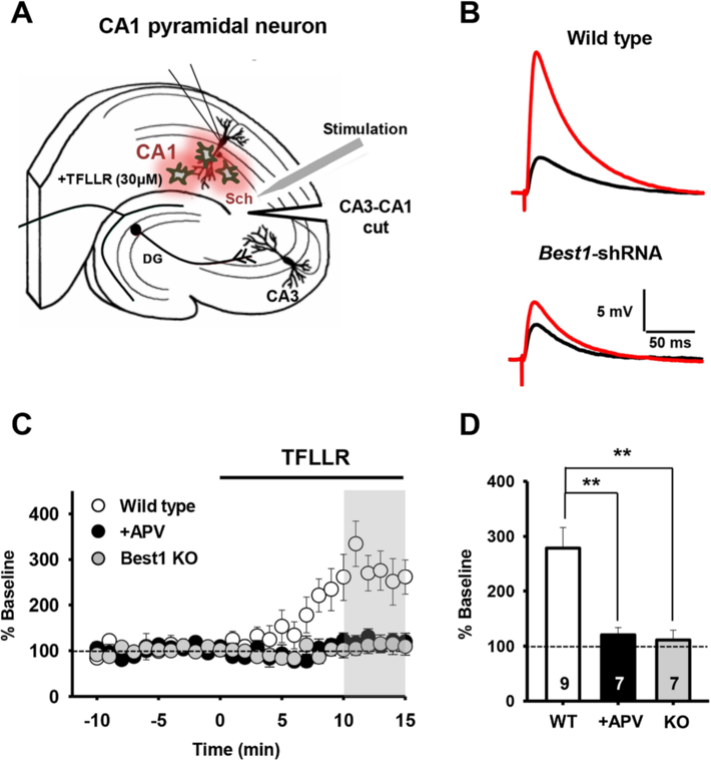
**Astrocytic glutamate released upon PAR1 activation induces NMDAR-dependent enhancement of basal EPSP responses at SC-CA1 synapses via the Best1 channel. A**, Schematic diagram showing whole-cell patch clamp measurement of eEPSPs upon Schaffer collateral (Sch) stimulation recorded from hippocampal CA1 pyramidal neurons (CA1). 30 μM of TFLLR was applied via the bath solution to induce Ca^2+^-dependent glutamate release from CA1 astrocytes. A surgical cut was made between CA3 and CA1 (CA3-CA1 cut). DG, dentate gyrus. **B**, Representative eEPSP traces recorded from CA1 neurons in naïve slices or slices expressing Best1-shRNA in astrocytes. Black, before TFLLR application; red, 15 min after TFLLR application. **C**, Amplitudes of eEPSPs from each recording were normalized to the mean amplitude of baseline period prior to TFLLR application and shown as averaged relative eEPSPs ± s.e.m. (% Baseline). Graphs represent eEPSP responses recorded from hippocampal slices of wild type mice treated with control solution (wild type, empty circles) or APV-containing solution (+APV, black circles), and hippocampal slices of Best1 knockout mice treated with control solution (Best1 KO, grey circles). **D**, Bar graph representing mean relative eEPSPs (mean ± s.e.m), analyzed during the period covered by the gray box in **C**. Bar colors correspond to colours in **C**. **, P < 0.01, one-way ANOVA with *post-hoc* test.

It is possible that basal synaptic transmission at SC-CA1 synapses is modulated by elevated synaptic glutamate level mediated by Best1 channel. We thus explored the exact effect of Best1-mediated astrocytic glutamate on synaptic transmission, by measuring evoked excitatory postsynaptic potentials (eEPSPs) at SC-CA1 synapses (Figure 2A,B). Our data showed that bath treatment of TFLLR for ~10 min induces an increase in the amplitude of basal eEPSPs (% baseline, 278.7 ± 37.5, n = 9 slices), and this potentiated synaptic responses were inhibited by pre-treatment with an antagonist for NMDAR [D-(2*R*)-amino-5-phosphonovaleric acid (D-APV), 50 μΜ; % baseline, 120.4 ± 13.4, n = 7 slices; Figure 2C,D]. This suggests that astrocytic glutamate is able to potentiate basal synaptic transmission via NMDAR-dependent pathways. TFLLR application also fails to induce an increase in eEPSP amplitudes from hippocampal slices of Best1 KO mice (% baseline, 111.1 ± 17.9, n = 7 slices; Figure 2C,D), indicating a requirement of the Best1 channel in PAR1-induced synaptic potentiation.

**Figure 3:**
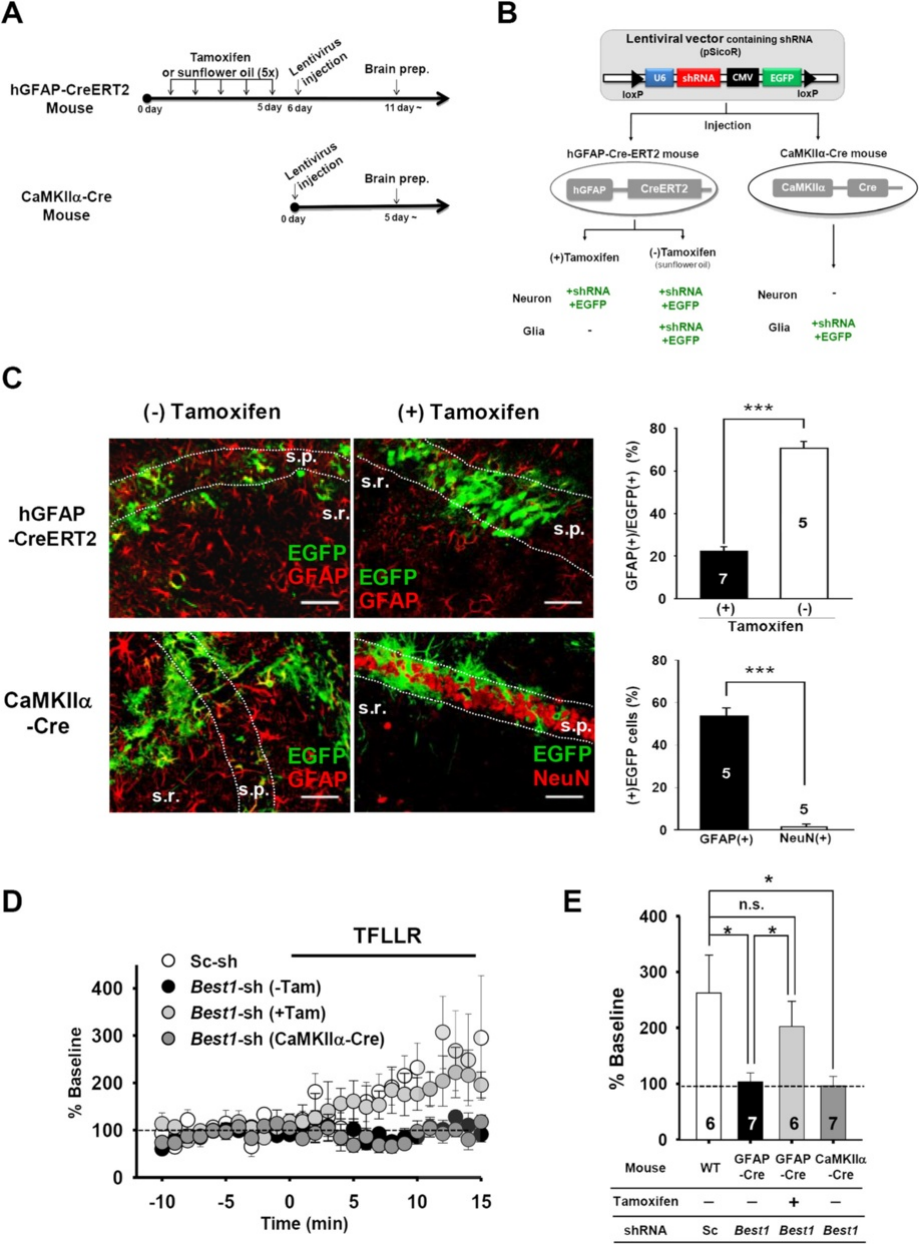
**Astrocytic Best1 is responsible for PAR1 activation-induced synaptic potentiation. A**, A schedule for tamoxifen and lentivirus injection into hGFAP-CreERT2, wild-type mice (naïve), or CaMKIIα-Cre mice. For acute Cre expression in hGFAP-CreERT2 mice, tamoxifen was pre-injected for 5 days before lentivirus injection. Sunflower oil, a control for tamoxifen. **B**, Diagram showing Cre-loxP regulation of shRNA expression in cell-type specific gene silencing system. In hGFAP-Cre-ERT2 mice, pretreatment of tamoxifen induces Cre expression, leading to neither shRNA nor EGFP expression in astrocytes through Cre/loxP-mediated deletion. Due to the same mechanism, CA1 pyramidal neurons cannot express both lentiviral shRNA and EGFP expression in CaMKIIα-Cre mice. **C**, ***Left***: Representative immunohistochemistry results showing Cre-dependent regulation of lentiviral shRNA expression. Cre-dependent shRNA expression in astrocytes or neurons was indicated by co-labeling of EGFP with GFAP or NeuN, respectively. s.p., stratum pyramidale; s.r., stratum radiatum. Scale bar = 50 μm. ***Right***: Bar graphs demonstrate the % of GFAP/EGFP double positive cells (hGFAP-CreERT2) or EGFP positive cells from GFAP- or NeuN positive cells (CaMKIIα-Cre) among total EGFP expressing cells. Mean (%) ± s.e.m. ***, p < 0.001, unpaired t-test. Numbers of counted slices at least from three mice were indicated within each bar. **D**, eEPSP responses from each recording shown as in Figure 2. Graphs represent averaged eEPSP responses (mean ± s.e.m.) recorded from loxP-floxed scrambled-shRNA expressing slices from all genotypes. (Sc-sh, empty circles), loxP-floxed *Best1*-shRNA expressing slices from hGFAP-Cre^ERT2^ without [*Best1*-shRNA (−Tam), black circles] or with tamoxifen [*Best1*-shRNA (+Tam), light grey circles], and loxP-floxed *Best1*-shRNA expressing slices from CaMKIIα-Cre mice [*Best1*-shRNA (CamKIIα-Cre), dark gray circles]. **E**, Bar graph representing mean eEPSPs (mean ± s.e.m), analyzed during the period covered by the gray box in **D**. Same bar colors as in **D**. *, P < 0.01, one-way ANOVA with *post-hoc* test. n.s., not significant.

To further investigate whether such synaptic potentiation is specifically mediated by Best1 expressed at astrocytes, we adapted a cell-type-specific gene silencing system to achieve both cell type-specific gene knockdown and recovery (Figure 3A,B; see also Methods) [25,29]. We injected a lentivirus containing loxP-floxed *Best1* short-hairpin RNA (shRNA) construct (pSicoR-*Best1*-shRNA) into the hippocampal CA1 area of transgenic mice expressing tamoxifen-inducible glial fibrillary acid protein (*Gfap*) promoter-driven Cre recombinase, Cre (hGFAP-Cre^ERT2^) or mice expressing Ca^2+^/calmodulin-dependent kinase II*α* promoter-driven Cre (CaMKIIα-Cre). Because loxP-floxed shRNA in the lentiviral construct can be cleaved by Cre expression in the pSicoR system [29], delivering a lentiviral particle containing pSicoR-*Best1*-shRNA into hGFAP-Cre^ERT2^ and CaMKIIα-Cre transgenic mice allowed us to achieve astrocyte- and neuron-specific recovery of Best1 expression, respectively (Figure 3C). Using this system, we were also able to examine the specific effect of astrocytic or neuronal Best1 on TFLLR-induced potentiation of basal synaptic transmission.

Our data showed that application of TFLLR to slices expressing *Best1*-shRNA globally in the CA1 region (mock-treated hGFAP-Cre^ERT2^ mice that were injected with pSicoR*-Best1*-shRNA) did not produce a TFLLR-induced increase in amplitude of eEPSPs (% baseline, 103.9 ± 15.7, n = 7 slices; Figure 3D,E), as observed from slices of Best1 KO mice (Figure 2C,D). However, when pSicoR-*Best1*-shRNA was injected into tamoxifen-treated hGFAP-Cre^ERT2^ mice (to recover Best1 expression specifically in astrocytes), TFLLR treatment was sufficient for inducing potentiated eEPSP responses (% baseline, 202.6 ± 45.0, n = 6 slices; Figure 3D,E). By contrast, an attempt to rescue Best1 expression in CA1 neurons, by injecting pSicoR-*Best1*-shRNA into CaMKIIα-Cre mice that express the Cre recombinase in CA1 pyramidal neurons [30], did not produce eEPSP potentiation upon TFLLR application (% baseline, 96.6 ± 16.9, n = 7 slices; Figure 3D,E). Together, these data indicate that the astrocyte-specific Best1 channel is required for enhanced basal synaptic transmission induced by astrocytic glutamate secretion upon PAR1 activation, implying a role of Best1-mediated glutamate in modulating synaptic activity.

### Best1-mediated astrocytic glutamate elevates synaptic glutamate

We next explored how astrocytic glutamate may enhance synaptic activity. Given the preferential distribution of astrocytic Best1 channels at microdomains that wrap around excitatory synaptic structures [10], the glutamate concentration at synaptic clefts may be directly affected by astrocytic glutamate release.

**Figure 4:**
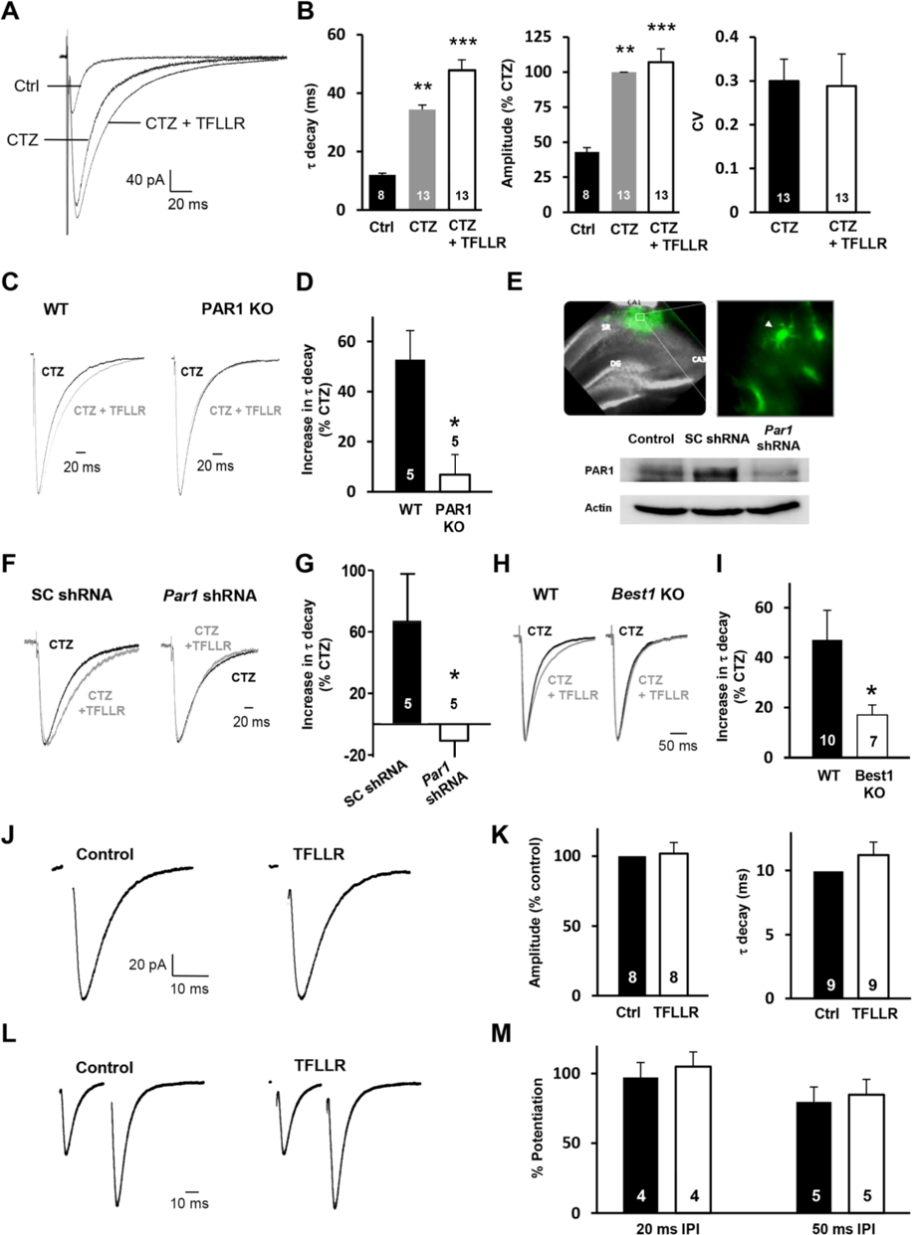
**Elevated synaptic glutamate by astrocytic glutamate release. A**, Representative AMPAR-EPSCs in the absence (Ctrl) or presence of CTZ (CTZ), and co-presence of CTZ and TFLLR (CTZ + TFLLR). **B**, Summary of decay kinetics (τ_decay_), amplitudes, and coefficient of variation (CV) of AMPAR-EPSCs (mean ± s.e.m). **, p < 0.01; ***, p < 0.001, one-way ANOVA with *post-hoc* test. **C**, Representative traces showing CTZ-induced changes in AMPAR-EPSCs in wild type (WT) or PAR1 knockout mice (PAR1 KO). **D,** Summary of increased decay kinetics (τ_decay_) of AMPAR-EPSCs by TFLLR application (mean ± s.e.m.). *, p < 0.05, unpaired t-test. **E**, ***Above***: representative fluorescence image showing expression of *Par1* shRNA in the hippocampal slice of CaMKIIα mice. SR, stratum radiatum. DG, dentate gyrus. The magnified view indicates nonneuronal expression of *Par1* shRNA (arrowhead). ***Below***: Western blot analysis showing PAR1 protein level in the naïve hippocampus (Control) or the hippocampus expressing scrambled-shRNA (SC shRNA) or *Par1* shRNA. **F**, Representative AMPAR-EPSCs, before (CTZ) and after TFLLR application (CTZ + TFLLR), in mice expressing SC or *Par1* shRNA. **G**, Bar graphs summarizing TFLLR-induced changes in τ_decay_ of CTZ-AMPAR-EPSC. Mean ± s.e.m. *, p < 0.05, unpaired t-test. **H**, Representative CTZ-AMPAR-EPSC traces in wild type (WT) or Best1 knockout mice (Best1 KO). **I,** Summary of TFLLR-induced increases in τ_decay_ of AMPAR-EPSCs (mean ± s.e.m.). *, p < 0.05, unpaired t-test. **J**, Representative AMPAR-EPSC traces before (Control) and after TFLLR application (TFLLR). **K**, Summary of amplitudes and τ_decay_ of the AMPAR-EPSCs. **L**, Representative traces showing paired-pulse facilitation (PPF) of AMPAR-EPSCs before (Control) and after TFLLR application (TFLLR). **M**, Summary of normalized PPF ratio (% potentiation, mean ± s.e.m.; 2^nd^ EPSC amplitude/1^st^ EPSC amplitude). IPI, inter-pulse interval. Numbers on the each bar graph indicate the number of tested slices from at least three mice.

At excitatory synapses, prolonged decay of evoked α-amino-3-hydroxy-5-methyl-4-isoxazolepropionic acid receptor (AMPAR)-mediated excitatory postsynaptic currents (EPSCs) in the presence of cyclothiazide (CTZ) is known to closely correlate with elevated glutamate concentration at the synaptic cleft [31-33]. By measuring changes in time constants of the mono-exponential decay (τ_decay_) of AMPAR-EPSCs in the presence of CTZ, we tested whether astrocytic glutamate is able to increase the synaptic glutamate level. In agreement with previous findings [32,33], our own application of CTZ (100 μM) significantly increased the peak amplitude and the τ_decay_ of AMPAR-EPSCs upon Schaffer collateral stimulation (Figure 4A,B). We found that astrocytic glutamate release triggered by TFLLR application further increased the τ_decay_ of CTZ-induced AMPAR-EPSCs with no significant change in amplitudes or the coefficient of variation (CV) of AMPAR-EPSC (Figure 4A,B).

We also showed that the increased τ_decay_ of CTZ-induced AMPAR-EPSCs by TFLLR application was mediated by selective activation of astrocytic PAR1, because TFLLR application in the presence of CTZ was unable to induce an increase in τ_decay_ of AMPAR-EPSCs measured from hippocampal slices of PAR1 knockout mice (PAR1 KO) (% increase in τ_decay_, wild type: 52.6 ± 11.6, n = 5 slices; PAR1 KO: 6.9 ± 7.9, n = 5 slices; Figure 4C,D) or in mice expressing astrocyte-specific *Par1*-shRNA (% increase in τ_decay_, scrambled shRNA: 68.1 ± 11.8, n = 5 slices; *Par1*-shRNA: 11.2 ± 28.5, n = 5 slices; Figure 4E-G). These results indicate that the prolonged activation of synaptic AMPARs is caused by an elevated synaptic glutamate level that results from astrocytic glutamate upon PAR1 activation. To examine whether Best1 channels are required for the TFLLR-induced increase in synaptic glutamate concentration, we compared the increased τ_decay_ of AMPAR-EPSCs by PAR1 activation in Best1 KO hippocampal slices with that in wild type slices. Our data showed that TFLLR application fails to induce an increase in τ_decay_ of AMPAR-EPSCs from hippocampal slices of Best1 KO mice as shown in slices of wild type mice (% increase in τ_decay_, wild type: 48.4 ± 10.9, n = 10 slices; Best1 KO: 15.6 ± 4.6, n = 7 slices; Figure 4H,I). Taken together, these results indicate that astrocytic glutamate released via Best1 channel targets synaptic clefts, causing an elevated synaptic glutamate concentration when combined with presynaptically released glutamate.

By contrast, we found no significant effect of TFLLR application on amplitudes and τ_decay_ of normal AMPAR-EPSCs in the absence of CTZ (% amplitude of AMPAR-EPSCs after TFLLR application = 102.0 ± 7.6, n = 8 slices; τ_decay_ after TFLLR application = 11.3 ± 1.0 ms, n = 9 slices; Figure 4J,K). In addition, presynaptic release properties were not affected by astrocyte-mediated increased synaptic glutamate, because PAR1 activation did not cause any significant alteration in the paired-pulse facilitation (PPF) of AMPAR-EPSCs (% potentiation; 20 ms IPI, control: 97.3 ± 10.8, TFLLR: 105.2 ± 10.6, n = 4 slices; 50 ms IPI, control: 79.7 ± 11.1, TFLLR: 85.1 ± 11.1, n = 5 slices; Figure 4L,M). These results suggest that elevated synaptic glutamate level by astrocytic glutamate affect neither postsynaptic AMPARs nor presynaptic glutamate receptors that can regulate the release probability. In support of this view, our previous study demonstrated unaltered frequencies and amplitudes of AMPAR-mediated miniature EPSCs (mEPSCs) upon PAR1 activation [25].

### Synaptic NMDARs are activated by Best1-mediated astrocytic glutamate

Because both presynaptic glutamate receptor and postsynaptic AMPAR are unaffected by synaptic glutamate elevation (Figure 4J-M), we next tested whether activity of synaptic NMDARs could be enhanced by astrocytic glutamate. We recorded NMDAR-mediated whole-cell currents at SC-CA1 synapses in the presence of low external Mg^2+^ (5 μM) and tetrodotoxin (1 μM), as previously used for showing that NMDAR-dependent whole-cell currents can be generated from CA1 neurons by Best1-mediated glutamate released upon PAR1 activation [10].

**Figure 5:**
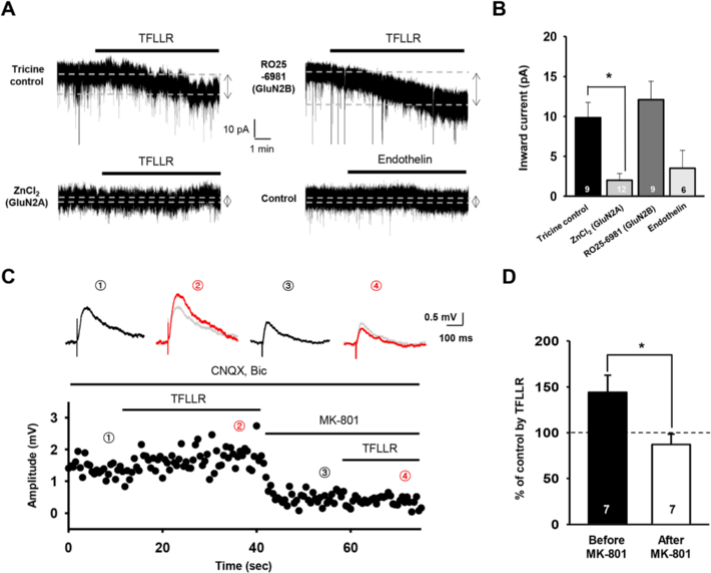
**Astrocytic glutamate released upon PAR1 activation targets GluN2A-containing synaptic NMDARs. A**, Representative recording traces indicating NMDAR-dependent whole-cell currents measured from a CA1 pyramidal neuron induced by the treatment with TFLLR(for PAR1) or endothelin (for endothelin receptor), in the presence of GluN2A (ZnCl_2_) or GluN2B-specific antagonist (RO25-6981). The arrow and dotted lines indicate the current responses before and after agonist treatments. **B**, Bar graph summarizing the averaged amplitudes of NMDAR-dependent currents induced by astrocytic glutamate (mean ± s.e.m). Numbers of tested slices from at least three independent mice are indicated within each bar. *, p < 0.05, unpaired two-tailed Student’s t-test. **C**, ***Upper***: representative NMDAR-eEPSP responses (isolated by co-application of 20 μM CNQX and 5 μM bicuculline) measured from CA1 pyramidal neuron, before (1) and after (2) TFLLR treatment in the absence of MK-801, and before (3) and after (4) TFLLR treatment in the presence of MK-801. Gray traces indicate NMDAR-eEPSP before TFLLR application in each experimental condition. ***Lower***: representative time course of the amplitude of NMDAR-eEPSPs from a single CA1 pyramidal neuron. Numbers (1) to (4) represent the same as those in the ***Upper*** section. **D**, Bar graph comparing normalized amplitudes of NMDAR-eEPSPs after TFLLR treatment with those before TFLLR treatment (% of control by TFLLR; mean ± s.e.m.). Numbers of tested slices from at least three independent mice are indicated within each bar. *, p < 0.05, unpaired two-tailed Student’s t-test.

Our data showed that PAR1 activation effectively increases the amplitude of NMDAR-mediated whole-cell currents from CA1 pyramidal neurons, whereas treatment with an agonist for other astrocytic native GPCR, endothelin, did not produce such currents (current amplitude, tricine control: 9.8 ± 1.9 pA, n = 9 slices; endothelin: 3.5 ± 2.3 pA, n = 6 slices; Figure 5A,B). A pretreatment with a GluN2A-specific antagonist (Zn^2+^; 250 nM), rather than with a GluN2B-specific antagonist (Ro25-6981; 2 μM) [34,35], was sufficient to eliminate the NMDAR-mediated currents induced by PAR1 activation (current amplitue, ZnCl_2_: 2.0 ± 0.8 pA, n = 12 slices; Ro25-6981: 12.1 ± 2.3 pA, n = 9 slices; Figure 5A,B), indicating that GluN2A-containing NMDARs is activated by increased synaptic glutamate. Given that major population of synaptic NMDARs contains GluN2A subunits in mature hippocampal synapses [35], our results suggest that astrocytic elevation of synaptic glutamate can activate synaptic NMDARs.

In order to directly examine whether the synaptic NMDAR activity can be enhanced by astrocytic glutamate, we isolated and measured NMDAR-mediated evoked EPSPs (NMDAR-EPSP: Figure 5C,D) at SC-CA1 synapses in the presence of 6-cyano-7-nitroquinoxaline-2,3-dione (CNQX) and bicuculline. After TFLLR application, the amplitude of evoked NMDAR-EPSPs was gradually increased (% baseline after TFLLR application: 144.2 ± 18.6%, n = 7 slices; Figure 5C,D), consistent with the idea that the activity of synaptic NMDAR is increased by astrocytic synaptic glutamate elevation. When opened synaptic NMDARs during synaptic transmission were fully blocked by bath application with MK-801 (50 μM; Figure 5C), we could not detect any increase in amplitude of NMDAR-EPSP on TFLLR application (% baseline after TFLLR application: 87.1 ± 11.3%, n = 7 slices; Figure 5C,D). Together with evidence for the direct activation of GluN2A-sensitive NMDAR by astrocytic glutamate (Figure 5A,B), these results support the notion that GluN2A-containing synaptic NMDARs are the major target of astrocytic glutamate, and our observation of NMDAR-dependent synaptic strengthening is resulted from increased activation of synaptic NMDARs by astrocytic synaptic glutamate elevation (Figure 2).

### Best1-mediated astrocytic glutamate modulates synaptic plasticity

**Figure 6:**
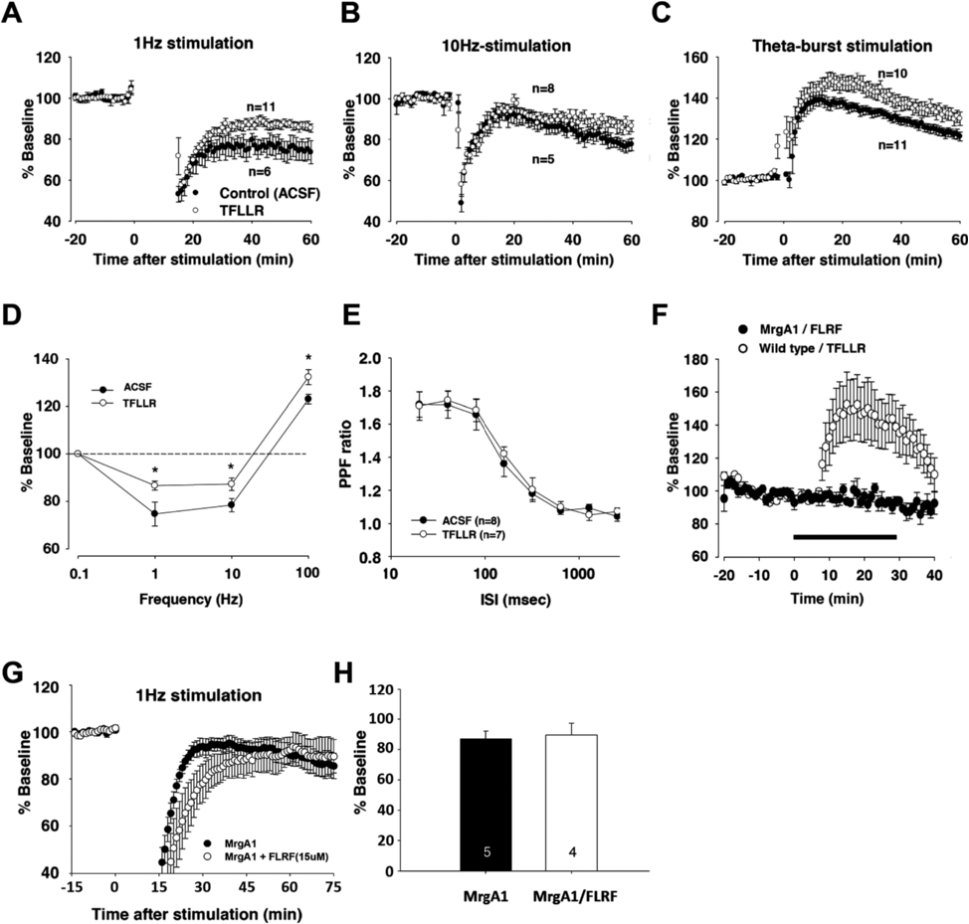
**Modulation of hippocampal synaptic plasticity by PAR1-induced glutamate release from astrocytes. A**-**C**, The magnitude of LTD induced by 1 Hz stimulation **(A)** and 10 Hz stimulation **(B)**, or LTP induced by theta-burst stimulation **(C)** in the absence (ACSF; black circles) or presence of TFLLR (TFLLR; white circles). Numbers of tested slices from at least three independent mice are indicated. **D**, Graph summarizing data in **A**-**C** by representing averaged fEPSP responses (mean ± s.e.m.) 50 ~ 60 min after application of each stimulation condition in the absence (ACSF; black circles) or presence of TFLLR (TFLLR; white circles). *, p < 0.05, unpaired Student’s t-test. **E**, Summary of paired-pulse facilitation (PPF) ratio of fEPSP responses in the absence (ACSF; black circles) or presence of TFLLR (TFLLR; white circles). ISI, inter-stimulus interval. The n numbers indicated for each group are the number of tested slices from at least five mice. **F**, Summary of basal fEPSP responses induced by treatment of either TFLLR to hippocampal slices of wild type mice (TFLLR) or FLRFamide to hippocampal slices of MrgA1 transgenic mice (MrgA1/FLRF) during the period indicated as a black line. **G**, Summary of LTD induction by applying 1 Hz (900 stimuli) to hippocampal slices of MrgA1 transgenic mice in the absence (MrgA1/ACSF; black circles) or presence of FLRFamide (MrgA1/FLRF; empty circles). **H**, Bar graph representing averaged % baseline of fEPSPs (mean ± s.e.m.) 50 ~ 60 min after stimulation. Numbers of tested slices from at least three independent mice are indicated within each bar. P = 0.780, unpaired two-tailed Student’s t-test.

We next asked whether long-term synaptic plasticity at SC-CA1 synapses could be modulated by astrocytic glutamate, because hippocampal synaptic plasticity are dependent on NMDAR-dependent Ca^2+^ signaling [36]. To examine the possible involvement of astrocytic glutamate in synaptic plasticity, we measured the effect of astrocytic PAR1 activation on the magnitude of long-term potentiation/depression (LTP/LTD) that were induced by electrical stimulation at various frequencies [1 Hz, 10 Hz, and theta-burst stimulation (TBS)] to Schaffer collateral fibers in hippocampal slices. Our field EPSP (fEPSP) recording measurements showed that the magnitude of LTD induced by either 1 Hz- or 10 Hz-stimulation in the presence of TFLLR was significantly lower than that in the absence of TFLLR (ACSF-treated) (% baseline, 1 Hz: 72.1 ± 5.5, n = 6 slices; 1 Hz + TFLLR: 84.8 ± 2.1, n = 11 slices; 10 Hz: 77.8 ± 2.7, n = 5 slices; 10 Hz + TFLLR: 86.9 ± 2.7, n = 8 slices Figure 6A,B,D). However, the magnitude of TBS-induced LTP in the presence of TFLLR was higher than that in the absence of TFLLR (% baseline, TBS: 122.8 ± 2.6, n = 11 slices; TBS + TFLLR: 131.9 ± 3.1, n = 10 slices; Figure 6C,D). Since the TFLLR treatment had no effect on presynaptic properties (Figures 4M, 6E), these altered changes in fEPSP responses by PAR1 activation appear to be mediated by postsynaptic mechanisms that include NMDAR-dependent signaling.

On the other hand, selective activation of astrocytic GPCR, Mas-related G-protein coupled receptor member A1 (MrgA1) by applying its agonist, FMRFamide peptide (FMRFa; ~ 30 min), to hippocampal slices of transgenic mice selectively expressing MrgA1 in astrocytes [16], was not efficient for altering fEPSP responses (Figure 6F). Moreover, in contrast to PAR1 activation, MrgA1 activation by FMRFa application could not alter the magnitude of LTD compared with control (% baseline, MrgA1: 86.9 ± 5.2, n = 5 slices; MrgA1 + FLRF: 89.5 ± 7.8, n = 4 slices; Figure 6G,H). These data are in agreement with previous reports showing that an increase in astrocytic Ca^2+^ by MrgA1 activation is not linked to the mechanisms responsible for triggering astrocytic glutamate release [15,16].

**Figure 7:**
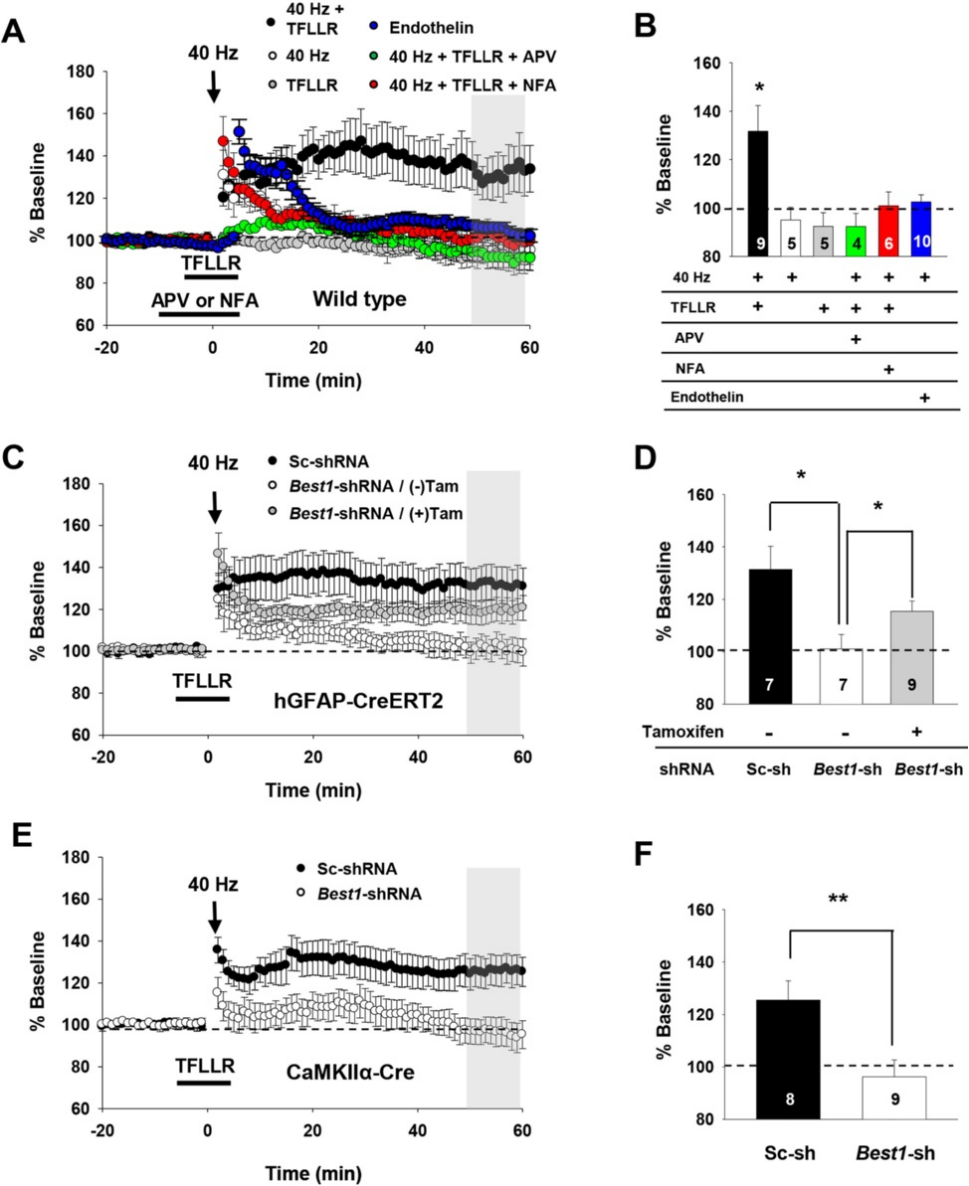
**A combination of subthreshold synaptic stimulation and Best1-mediated astrocytic glutamate produces NMDAR-dependent LTP at SC-CA1 synapses. A**, Summary of LTP recordings in hippocampal slices when subthreshold stimulation (40 Hz) with TFLLR (40 Hz + TFLLR; black circles), 40 Hz alone (40 Hz; white circles), TFLLR alone (TFLLR; gray circles), 40 Hz with TFLLR in the presence of APV (40 Hz + TFLLR + APV; green circles) or niflumic acid (40 Hz + TFLLR + NFA; red circles), and 40 Hz with endothelin (Endothelin; blue circles) was applied. **B**, Bar graph representing the averaged % baseline of fEPSPs (mean ± s.e.m.) over the time period indicated by the gray bar in **A**. *, p < 0.05, one-way ANOVA with *post-hoc* test. Same colors as **A**. Numbers of tested slices from at least three mice are indicated within each bar. **C,** Summary of LTP recording from loxP-floxed scrambled-shRNA expressing hippocampal slices (Sc-shRNA, black circles), loxP-floxed Best1-shRNA expressing slices from hGFAP-Cre^ERT2^ mice without [*Best1*-shRNA (−Tam), white circles] or with tamoxifen pretreatment [*Best1*-shRNA (+Tam), gray circles]. **D**, Bar graph representing the averaged % baseline of fEPSPs (mean ± s.e.m.) over the time period indicated by the gray bar in **C**. *, p < 0.05, one-way ANOVA with *post-hoc* test. Same colors as **C**. Numbers of tested slices from at least three mice are indicated within each bar. **E**, Summary of LTP recording from Sc-shRNA expressing hippocampal slices (black circles) or *Best1*-shRNA expressing slices from CaMKIIα-Cre mice (white circles). **F**, Bar graph representing the averaged % baseline of fEPSPs (mean ± s.e.m.) over the time period indicated by the gray bar in **E**. *, p < 0.05, unpaired t-test. Same colors as **E**. Numbers of tested slices from at least independent mice are indicated within each bar.

We next investigated whether an increased NMDAR activity by astrocytic glutamate might affect LTP induction. Due to preferential targeting of astrocytic glutamate to synaptic NMDARs, we predicted that normal NMDAR-dependent LTP could be induced when subthreshold electrical stimulation for LTP induction is combined with astrocytic synaptic glutamate elevation. We found that neither subthreshold electrical stimulation (40 Hz, 10 stimuli: Figure 7A) nor a short period of TFLLR application (less than ~7 min) to the hippocampal slices was sufficient for inducing synaptic potentiation when treated alone (% baseline, 40 Hz alone: 95.0 ± 5.3, n = 5 slices; TFLLR alone: 92.4 ± 5.7, n = 5 slices; Figure 7A,B). However, combined application of subthreshold stimulation with TFLLR (40 Hz + TFLLR) was effective in inducing LTP that was sensitive to pretreatment with APV or an anion channel blocker, niflumic acid (NFA; 100 μM) (% baseline, 40 Hz + TFLLR: 131.65 ± 10.6, n = 9 slices; 40 Hz + TFLLR + NFA: 101.0 ± 5.6, n = 6 slices; Figure 7A,B). To test whether this LTP requires Best1-mediated glutamate, we measured 40 Hz + TFLLR-induced LTP responses from hippocampal slices, of which Best1 gene expression was manipulated in a cell-type specific manner as performed previously (see Figure 3). We found that 40 Hz + TFLLR-induced LTP is significantly prevented by general expression of Best1 shRNA in the hippocampal CA1 area (slices from mock-treated hGFAP-Cre^ERT2^ mice injected with pSicoR-Best1-shRNA), whereas hippocampal slices with astrocyte-specific rescue of Best1 expression (from tamoxifen-treated hGFAP-Cre^ERT2^ mice injected with pSicoR-Best1-shRNA) showed 40 Hz + TFLLR-induced LTP comparable to that from the same transgenic mice injected with scrambled-shRNA (Sc-shRNA) (% baseline, Sc-shRNA: 131.3 ± 8.8, n = 7 slices; Best1-shRNA w/o taxmoxifen: 101.1 ± 5.5, n = 7 slices; Best1-shRNA w/tamoxifen: 115.43 ± 3.9, n = 9 slices; Figure 7C,D). These results indicate that glutamate released via the Best1 channel in CA1 astrocytes is required for NMDAR-dependent LTP induced by the 40 Hz + TFLLR stimulation. In accordance with these findings, neuron-specific rescue of Best1 expression (CaMKIIα-Cre mice with pSicoR-Best1–shRNA) failed to induce LTP with the 40 Hz + TFLLR stimulation (% baseline, Sc-shRNA: 125.5 ± 7.2, n = 8 slices; Best1-shRNA: 96.2 ± 6.4, n = 9 slices; Figure 7E,F). As a whole, our data show that glutamate released via astrocytic Best1 plays a significant role in modulating synaptic plasticity by lowering the threshold for LTP induction.

## Discussion

**Figure 8:**
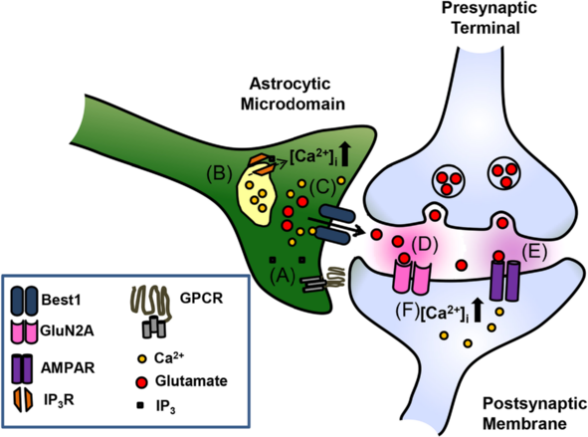
**Schematic diagram of proposed physiological roles for Ca** ^**2+**^ **-dependent astrocytic glutamate release via Best1 channel in synaptic plasticity.** We propose a model for the modulation of synaptic function via the action of astrocytic Best1-mediated glutamate on postsynaptic NMDARs. **A**, Astrocytic GPCR is activated by neurotransmitter or neuronal factors. **B**, Downstream signaling pathways induce release of Ca^2+^ from internal stores, resulting in an increase in intracellular Ca^2+^ concentration ([Ca^2+^]_i_) at microdomains that wrap around synaptic terminals. **C**, Increased microdomain Ca^2+^ activates the Ca^2+^-dependent glutamate-permeable anion channel (Best1), leading to glutamate efflux from astrocytes. **D**, Best1-mediated glutamate binds to synaptically localized NMDAR (GluN2A). **E**, Glutamate released from presynaptic neuron activates AMPAR, which causes postsynaptic depolarization. **F**, When postsynaptic depolarization resulting from synaptic activity is accompanied by the binding of astrocytic glutamate to synaptic NMDARs, Ca^2+^ influx to postsynaptic sites is increased, resulting in the enhanced Ca^2+^-dependent signaling required for increasing synaptic strength.

Our study reveals the physiological potential for receptor-mediated and channel-dependent astrocytic glutamate release in synaptic function. This action is mediated by the mechanism initiated by astrocytic GPCR activation that can lead to glutamate secretion via the Ca^2+^-activated anion channel (Best1) in astrocytes (Figure 8). We provide direct evidence that GluN2A-containing postsynaptic NMDAR is the main glutamate receptor sensing astrocytic glutamates or elevated synaptic glutamate levels (Figure 5A,B). Because activation of GluN2-containing NMDARs is dependent on the depolarization-induced removal of Mg^2+^ block [37], binding of astrocytic glutamate to synaptic NMDARs may permit increased NMDAR opening when coincident postsynaptic depolarization is accompanied during spontaneous or evoked synaptic transmission (Figure 8). Increased NMDAR activation from the participation of astrocytic glutamate would lead to elevated Ca^2+^ influx into postsynaptic sites and in turn trigger diverse signaling pathways responsible for synaptic strengthening (Figure 8).

Consistent with previous reports [14,15,17,38], activation of astrocytic GPCRs, with the exception of PAR1, was not sufficient to trigger glutamate release from astrocytes (Figures 6F and 7A), supporting the idea that glutamate released upon astrocytic PAR1 activation has a specific modulatory role in synaptic function. The reason that PAR1 is more effective than other GPCRs for inducing glutamate release from astrocytes remains to be determined, but it is possible that activation of MrgA1 or other GPCRs only causes an insufficient Ca^2+^ levels at microdomains for triggering Ca^2+^-dependent glutamate release mechanisms [38]. On the other hand, either MrgA1 or other GPCRs may not be associated with the astrocytic molecular mechanisms responsible for glutamate release, whereas PAR1 is linked to multiple channel-mediated glutamate release mechanisms such as G protein-dependent opening of the two-pore K^+^ channel (TREK-1) and Ca^2+^-dependent opening of the Best1 channel [10]. Thus, if PAR1 activation occurs during the process of synaptic plasticity, astrocytic glutamate released through channel-mediated mechanisms may play a significant role in modulating hippocampal synaptic plasticity and cognitive functions. In support of this idea, it has been shown that activation of PAR1 by thrombin (a native PAR1 agonist) or TFLLR modulates LTP formation at hippocampal synapses [27,39], and that a reduction in NMDAR-dependent hippocampal LTP and contextual fear memory were observed in PAR1 deficient mice [27]. How astrocytic PAR1 is activated during the process of synaptic plasticity and memory formation is still unclear, but we suggest that astrocytic PAR1 might be activated by endogenous PAR1 agonists that are generated from secreted proteases or their byproducts in an activity-dependent manner. For example, plasmin, which is cleaved from plasminogen by the extracellular tissue plasminogen activator (tPA), can act as a native agonist for PAR1 in the hippocampus [24], and activity-dependent secretion of tPA from postsynaptic dendrites [40,41] is required for inducing late-phase LTP (L-LTP) at hippocampal synapses [42,43].

Our study has also provided evidence for the involvement of PAR1-triggered astrocytic glutamate in synaptic function by showing that the Best1 channel is required for synaptic NMDAR activation. Ultrastructural analyses showed that Best1 channels are preferentially expressed at the membrane of astrocytic microdomains around synaptic terminals in the hippocampus (Figure 1), proposing a possible influence of Best1-mediated glutamate on the synaptic glutamate level [10]. Consistent with this, we have shown that PAR1 activation prolongs the CTZ-induced AMPAR-EPSC response, an action indicative of increased synaptic glutamate levels, and that this prolonged AMPAR-EPSC response was dependent on Best1 expression (Figure 4). Because the amount of glutamate released through the Best1 channel was much lower (10^−5^ ~ 10^−6^ M; Figure 1E-G) than that from presynaptically released glutamate (~10^−3^ M), a subtle increase in synaptic glutamate levels by PAR1 activation thus had no significant effect on synaptic glutamate receptors, other than NMDARs [2,20,39], as shown by the unchanged presynaptic release probability and AMPAR-mediated postsynaptic activity (Figure 4J-M). Thus, we suggest that enhanced NMDAR-dependent signaling by receptor-mediated astrocytic glutamate release can produce potentiation of AMPAR-mediated synaptic transmission (Figure 2), because AMPAR expression at the postsynaptic membrane could be elevated by activation of NMDAR-dependent signaling [44,45]. However, our study do not exclude the possibility that astrocytic modulation of synaptic NMDAR activity and neurotransmission is mediated by other active substances such as D-serine [46,47], which might be released through Best1 channels in astrocytes. Further studies are required for clarifying the role of astrocytes in modulating neural circuits and behaviors by dissecting the exact functions of Best1 channels in neurotransmission.

## Conclusions

In summary, our present study reveals that astrocytes can play a significant role in regulating neural synaptic functions, by releasing glutamate via Ca^2+^-activated anion channels opened by the activation of receptor-mediated signaling. The models proposed and the tools developed in this study aim to improve our understanding of the physiological role of multiple glutamate release mechanisms, along with their significance in cognitive functions.

## Methods

### Ethical approval

All animal protocols were performed in accordance with the institutional guideline of Korea institute of Science and Technology (KIST; Seoul, Korea).

### FRET-based glutamate imaging

The amount of released extracellular glutamate was represented by the ratio between the emission intensity of CFP and YFP (CFP/YFP), which was divided by baseline CFP/YFP ratio (relative CFP/YFP ratio). Adenovirus containing pDisplay-GluSnFR [28] was injected into the hippocampal CA1 region. After one week, FRET imaging in acute hippocampal slices was performed under a microscope (BX50WI; Olympus) equipped with a xenon lamp fitted with a 436/20-excitation filter (D436/20x filter; Chroma). The emission beam was split using a Dual-View (Optical Insights) fitted with a CFP/YFP filter set (OI-05-EX), and recorded with an iXon EMCCD camera (Andor). Imaging Workbench software (INDEC BioSystems) was used for image acquisition and offline image analysis. TFLLR puffs (TFLLR-NH_2_; Peptron, Korea; 500 μM) were applied using picospritzer-assisted positive pressure (~100 ms).

### Production of shRNA containing lentivirus and delivery into mouse hippocampus

Scrambled shRNA, mBest1-shRNA, and *Par1*-shRNA were inserted into pSicoR lentiviral vector (provided by Dr. T. Jacks through Addgene Inc.; [29] as previously described [8]. For shRNA expression in hippocampal CA1 region, lentivirus (produced by Macrogen, Korea) was introduced into the hippocampal CA1 region by the stereotaxic surgery method [48]. hGFAP-CreERT2 transgenic mice were provided by Dr. Ken McCarthy. CaMKIIα-Cre transgenic mice were purchased from The Jackson Laboratory. hGFAP-CreERT2 mice were used at the age of 7 weeks for tamoxifen or sunflower oil injection (intraperitoneal injection, once per day for 5 days). The lentivirus carrying shRNA was injected 1 day after the fifth day injection. CaMKIIα-Cre mice were used at 8 weeks of age for virus injection and were used at around 9 weeks for electrophysiological recordings. Only male mice were used throughout the study. Injected mice were sacrificed for electrophysiological recordings at 8 ~ 9 weeks of age.

### Slice preparation and electrophysiology

Horizontal or transverse mouse brain slices (300 ~ 400 μm) containing hippocampus were acutely prepared as previously described [20]. Prepared slices were left to recover for at least 1 hour before recording, in oxygenated (95% O_2_ and 5% CO_2_) artificial cerebrospinal fluid (ACSF; in mM, 130 NaCl, 24 NaHCO_3_, 3.5 KCl, 1.25 NaH_2_PO_4_, 1 CaCl_2_, 3 MgCl_2_ and 10 glucose, pH 7.4; room temperature). The standard ACSF recording solution was composed of (mM): 130 NaCl, 24 NaHCO_3_, 3.5 KCl, 1.25 NaH_2_PO_4_, 1.5 CaCl_2_, 1.5 MgCl_2_ and 10 glucose saturated with 95% O_2_ and 5% CO_2_, at pH 7.4. To block the effect of neuronal spontaneous activity on astrocytes, TTX (0.5 μM; Tocris Bioscience) was added into the ACSF. Experiments with a holding current of more than −100 pA or in which there was a change in input resistance >30% of the control were rejected. Recordings were obtained using Axopatch 200A (Axon Instruments) and were filtered at 2 kHz. mEPSC recordings were digitized at 10 kHz and analyzed using pCLAMP 9 software (Axon Instruments) and Mini Analysis Program software (Synaptosoft) as previously described [20]. Whole-cell recordings from CA1 neuron, mEPSC recordings, and eEPSP recordings were carried out as previously described [20]. For making Zn-included ACSF solution, 250 nM ZnCl_2_ was used in 10 mM Tricine with the relation [Zinc]_free_ = [Zinc]_applied_/200 as previously described [34,35]. CA1 fEPSPs were evoked by Schaffer collateral using a bipolar electrode and quantified as the initial slope of fEPSP as previously described [49]. To examine the effect of TFLLR on synaptic plasticity at different stimulation frequencies, slices were perfused with TFLLR (30 μM) ~15 min before stimulation. Electrical stimulations were given as 1 Hz (900 sec), 10 Hz (90 sec), and theta-burst stimulation (consisting of four trains containing ten bursts (each with four pulses at 100 Hz) of stimuli delivered every 200 msec). For 40 Hz + TFLLR-induced LTP induction experiments, TFLLR (30 μM) was applied ~8 min before 40 Hz stimulation and washed out 2 min after stimulation. APV (Tocris Bioscience; 50 μM), niflumic acid (Sigma; 100 μM) was co-treated with TFLLR.

### Immunohistochemistry

Adult mice were deeply anesthetized with 2% avertin and perfused with 0.1 M PBS followed by 4% paraformaldehyde. Brains were postfixed in 4% paraformaldehyde at 4°C for 24 hr and 30% sucrose at 4°C for 48 hr. Brains were then cut into 30 μm coronal cryosections. Sections were blocked in 0.1 M PBS containing 0.3% Triton X-100 (Sigma) and 2% Donkey Serum (GeneTex) for 30 min at room temperature. Primary antibody was then applied at the appropriate dilution and incubated overnight at 4°C. Sections were then washed three times in 0.1 M PBS and incubated in secondary antibody for 2 h. After three rinses in 0.1 M PBS and DAPI staining at 1:1000 (Pierce), the sections were mounted on polysine microscopic glass slides (Thermo Scientific). Images were acquired using a Nikon A1R confocal microscope.

### Electron microscopy

Animals were deeply anesthetized with sodium pentobarbital (80 mg/kg, i.p.) and perfused transcardially with heparinized normal saline (10 ml for mouse and 100 ml for rat), followed by a freshly prepared mixture (50 ml for mouse and 500 ml for rat) of 4% paraformaldehyde and 0.01% glutaraldehyde in 0.1 M phosphate buffer (PB), pH 7.4. Hippocampus was removed and postfixed in the same fixative for 2 hours at 4°C. Sagittal sections (60 μm) were cut with a vibratome and cryoprotected in 30% sucrose in PB overnight at 4°C. Sections were frozen on dry ice for 20 minutes, thawed in phosphate-buffered saline (PBS; 0.01 M, pH 7.2) to enhance penetration. They were pretreated with 1% sodium borohydride for 30 minutes to quench glutaraldehyde and then blocked with 3% H_2_O_2_ for 10 minutes to suppress endogenous peroxidases, and with 10% Normal Donkey Serum (NDS, Jackson ImmunoResearch, West Grove, PA) for 30 minutes to mask secondary antibody binding sites. For double immunostaining of GFP and Best1, sections of hippocampus pretreated as above were incubated overnight in a mixture of mouse anti-GFP (1:400, MAB3580, Millipore, Temecula, CA) and rabbit anti-Best1 (1:200) antibodies. After rinsing in PBS, sections were incubated with a mixture of biotinylated donkey anti-mouse (1:200, Jackson ImmunoResearch) and 1 nm gold-conjugated donkey anti-rabbit (1:50, EMS, Hatfield, PA) antibodies for 2–3 hours. The sections were postfixed with 1% glutaraldehyde in PB for 10 minutes, rinsed in PB several times, incubated for 4 minutes with HQ silver enhancement solution (Nanoprobes, Yaphank, NY), and rinsed in 0.1 M sodium acetate and PB. Serially cut thin sections were collected on Formvar-coated single slot nickel grids and stained with uranyl acetate and lead citrate. Grids were examined on a Hitachi H-7500 electron microscope (Hitachi, Tokyo, Japan) at 80 kV accelerating voltage. Images were captured with Digital Montage software driving a MultiScan cooled CCD camera (ES1000W, Gatan, Pleasanton, CA) attached to the microscope and saved as TIFF files.

### Statistical analyses

All data were shown as mean ± standard error of the mean (s.e.m.). Statistical analyses were performed by using Sigma Plot software (ver. 10.0; Systat Software Inc., San Jose, CA). Detailed information of the statistical tests was indicated in the figure legends.
