# Correction to: Channel-mediated astrocytic glutamate modulates hippocampal synaptic plasticity by activating postsynaptic NMDA receptors

**DOI:** 10.1186/s13041-021-00811-9

**Published:** 2021-06-29

**Authors:** Hyungju Park, Kyung-Seok Han, Jinsoo Seo, Jaekwang Lee, Shashank M. Dravid, Junsung Woo, Heejung Chun, Sukhee Cho, Jin Young Bae, Heeyoung An, Woohyun Koh, Bo-Eun Yoon, Rolando Berlinguer-Palmini, Guido Mannaioni, Stephen F. Traynelis, Yong Chul Bae, Se-Young Choi, C. Justin Lee

**Affiliations:** 1 Center for Neural Science, Korea Institute of Science and Technology (KIST), Seoul, Korea; 2 Neuroscience Program, University of Science and Technology (UST), Daejeon, Korea; 3 Department of Physiology and Dental Research Institute, Seoul National University School of Dentistry, Seoul, Korea; 4 Department of Pharmacology, Creighton University, Omaha, NE USA; 5 Department of Oral Anatomy and Neurobiology, School of Dentistry, Kyungpook National University, Daegu, Korea; 6 KU-KIST Graduate School of Converging Science and Technology, Korea University, 145 Anam-ro, Seongbuk-gu, Seoul, 02841 Korea; 7 Department of Nanobiomedical Science, Dankook University, Cheonan, Korea; 8 School of Electrical and Electronic Engineering, Institute of Neuroscience, Newcastle University, Newcastle upon Tyne, UK; 9 Department of Pharmacology, University of Florence, Florence, Italy; 10 Department of Pharmacology, Emory University, Atlanta, GA USA

## Correction to: Mol Brain (2015) 8:7 10.1186/s13041-015-0097-y

Following publication of the original article [1], the authors would like to correct incomplete presentation of affilition 6. The corrected and complete presentation of affiliation 6 is:

^6^KU-KIST Graduate School of Converging Science and Technology, Korea University, 145 Anam-ro, Seongbuk-gu, Seoul, 02841, Korea.

The affiliation list has been updated in this Correction article and the original article [1] has been corrected.
